# Characterization of the *Escherichia coli* Antifungal Protein PPEBL21

**DOI:** 10.1155/2010/196363

**Published:** 2010-05-17

**Authors:** V. Yadav, R. Mandhan, M. Kumar, J. Gupta, G. L. Sharma

**Affiliations:** ^1^National Institute for Health, Rockville Pike, Bethesda, MD 20892, USA; ^2^Department of Biotechnology, Kurukshetra University, Kurukshetra 136119, India; ^3^Institute of Genomics and Integrative Biology, Mall Road, Delhi 110007, India; ^4^Research Institute of the McGill University Health Centre, Montréal, QC, Canada H3G 1A4

## Abstract

An antifungal protein isolated from *Escherichia coli* BL21 (PPEBL21) and predicted to be alcohol dehydrogenase (ADH) was subjected to biological characterization. The PPEBL21, indeed, demonstrated propionaldehyde-specific ADH activity. The Km and Vmax of PPEBL21 were found to be 644.8 *μ*M and 1.2 U/mg, respectively. In-gel activity assay also showed that PPEBL21 was a propionaldehyde-specific ADH. The pI of PPEBL21 was observed to be 7.8. PPEBL21 was found to be stable up to a temperature of 40°C with optimum activity at pH 7.5. The decrease in pH decreased the activity of PPEBL21. These results suggested that PPEBL21 having alcohol dehydrogenase activity and stability at significantly high temperature might be an important lead antifungal molecule. Experiments were performed to identify the possible target of PPEBL21 in the pathogen *A. fumigatus*. Results revealed that PPEBL21 inhibited completely the expression of a 16 kDa protein in *A. fumigatus*. The 16 kDa protein of *A. fumigatus* targeted by PPEBL21 was identified as a hypothetical protein by peptide mass fingerprinting. It is thus hypothesized that a 16 kDa factor is essentially required by *A. fumigatus* for survival and its impaired synthesis due to treatment with PPEBL21 may lead to the death of pathogen.

## 1. Introduction

The need for developing new, safe and more effective antifungal drugs has been a major challenge today, especially with alarming increase in the incidence of opportunistic life-threatening fungal infections due to variety of factors including indiscriminate use of antibiotics, immunosuppressive therapies, blood transfusions, organ transplantation, and underlying diseases such as aplastic anemia, AIDS, chronic granulomatous disease, and Job's syndrome [1]. The history of new drug discovery processes has shown that novel skeletons with antimycotic properties have, in the majority of cases, come from natural sources [2]. There have been efforts which involved the screening of plants and microorganisms for antimycotic properties [3–5]. However, the progress on the search for new, broad-spectrum antifungal compounds with greater potency has been very slow [6]. One reason for the slow progress compared to antibacterials has been that, like mammalian cells, fungi are also eukaryotes and therefore agents that inhibit protein, RNA, or DNA biosynthesis in fungi have a greater potential for toxicity to the host as well [7]. Another reason has been that, until recently, the incidence of life threatening fungal infections was perceived as being too low to warrant aggressive research aiming at developing ideal antifungal formulations [8]. 

 Under a research programme on new drug development, we evaluated a panel of bacteria for antimycotic potential [9]. It was observed that *Escherichia coli* strain BL21 possessed antifungal properties and its activity was associated with a 39.30 kDa protein synthesized by *E. coli *BL21 [10]. The primary amino acid structure of 39.30 kDa protein of *E. coli *BL21 (PPEBL21) did not resemble that of antifungal proteins described so far and therefore, it could be an important lead from different class of molecules. The present study was undertaken to characterize the PPEBL21 partially. Attempts were also made to identify the target(s) of PPEBL21 in *A. fumigatus *that might play a role in the survival of the pathogen.

## 2. Materials and Methods

### 2.1. Bacterial Strain

*Escherichia coli* BL21 (MTCCB 1678) was procured from Institute of Microbial Technology, Chandigarh (India). The *E. coli* was cultured in LB Broth for 3 days at 37°C in a shaker incubator. The cells were counted by the turbidity method and used for performing various experiments. 

### 2.2. Pathogens

Pathogenic strains of *A. fumigatus* were obtained from Microbiology Department of Vallabhbhai Patel Chest Institute, Delhi, India and cultured in Sabouraud dextrose agar for 4 days. The plates were used as the source of spores for performing experiments.

### 2.3. Purification of PPEBL21

An activity guided purification of antifungal molecule from *E. coli* lysate was performed. The lysate prepared from BL21 strain of *E. coli* was subjected to fractionation by ion exchange chromatography [9]. The proteins recovered in an active ion exchange chromatographic fraction (F III) were subfractionated by gel filtration. An amount of 3.0 mg of F III proteins was applied onto a Sephadex G 100 column (1.5 × 75.0 cm) and eluted with 10 mM Tris-HCl buffer (pH 7.5) at a flow rate of 0.5 ml min^−1^ to obtain five subfractions (SF 1 to SF 5). The active SF 3 was further examined for purity by polyacrylamide gel electrophoresis (PAGE) and HPLC using C8 column. The purified active protein of *E. coli *(PPEBL21) was subjected to detailed biological characterization.

### 2.4. Elucidation of Isoelectric Point of PPEBL21

The isoelectric point (pI) of PPEBL21 was determined by using modified method described by Towbin et al. [11]. Prior to isoelectric focusing, the protein sample was prepared by 2D clean up kit (Amersham Biosciences) as recommended by suppliers. An amount of 100 *μ*g of protein was reconstituted in 50 *μ*l of IEF-loading buffer (8 mM urea, 50 mM DTT, 2% Triton X-100, and 1.2% ampholyte pH 3–10). IPG strips were rehydrated with sample overnight and subjected to first-dimension electrophoresis on IEF cell (BioRad, CA, USA) by using standard program recommended by the manufacturer. Second-dimension electrophoresis was done on 12.5% SDS-PAGE in Mini Protean assembly (BioRad, CA, USA). The gel was silver stained for further analysis.

### 2.5. Biochemical Analysis of PPEBL21

The analysis of N-Terminal amino acid sequence and peptide mass fingerprinting revealed that the PPEBL21 could be an alcohol dehydrogenase (ADH) [10]. Therefore, PPEBL21 in native form was studied for its enzymatic properties.

#### 2.5.1. ADH Activity

An amount of 10 *μ*g of PPEBL21 was dissolved in 100 *μ*l of sodium pyrophosphate buffer, pH 7.5. The protein solution in microwell of a plate was incubated with 10 mM of ADH-specific aldehydes (such as acetaldehyde, propionaldehyde, formaldehyde, and benzaldehyde all from M/s Sigma Chemical Company, USA) and 25 mM NADH solution. The wells were examined for the development of colour, and the OD at 340 nm of the solution was measured from 1 to 20 minutes using a microwell plate reader (Spectra Max Plus, Molecular Devices, USA). The OD was plotted against time to determine the enzyme activity.

#### 2.5.2. In-Gel ADH Activity Assay

The in-gel enzyme activity of PPEBL21 was determined by the method of Birken and Pisano [12]. PPEBL21 was run on 12.5% native gel and treated with staining mixture which was consisted of 50 ml of 50 mM sodium pyrophosphate buffer pH 7.5, 1.0 ml of 10 mg/ml NADH (Sigma Chemical Company, USA) solution, 50 ml of 100.0 mM of aldehyde (acetaldehyde, propionaldehyde, formaldehyde or benzaldehyde), 1.0 ml of 10 mg/ml nitro blue tetrazolium (Sigma Chemical Company, USA), and 0.4 ml of 50 mg/ml of phenazine methosulfate (Sigma Chemical Company, USA) solution. Gel was incubated in staining solution at 37°C until clear zone of activity was optimally developed. The reaction was stopped by adding water to the gel. The gel was further washed with water three times and stored in 7% acetic acid.

#### 2.5.3. Enzyme Kinetics

The ADH enzyme kinetics of PPEBL21 was studied using the basic method of Vallee and Hoch [13]. ADH activity was assayed in 96 well plates containing 50 mM sodium pyrophosphate buffer pH 7.5, 25 mM NADH, and 10.0 *μ*g of PPEBL21. The standard ADH was used in control wells. The reaction was started by the addition of different concentrations of propionaldehyde as the enzyme substrate. The rate of reaction was monitored using a microwell plate reader (Spectra Max Plus, Molecular Devices, USA) in kinetic mode. Specific activities were expressed as the rate of NAD formed/min/mg protein. Vmax and Km of PPEBL21 were calculated by using software (Sigma Plot 8.0). 

### 2.6. Stability of PPEBL21

The stability of protein at different temperatures ranging from 4 to 100°C was determined by incubating PPEBL21 in 10 mM Tris-HCl buffer (pH 7.5) for 20 minutes. The solution of protein was cooled to 4°C and examined for anti-*Aspergillus* activity by percentage spore germination inhibition (PSGI) assay [4]. The stability of PPEBL21 under acidic and alkaline conditions was tested by using citrate phosphate buffer (pH 2.5 to 8.0) and Tris-HCl buffer (pH 7.5 to 10.5). PPEBL21 was incubated in each buffer at various pHs at 4°C for 20 minutes. The anti-*Aspergillus* activity was assayed as per the method of Rajesh and Sharma [4] after the pH of each PPEBL21 solution was readjusted to 7.5.

### 2.7. Identification of Gene Products/Protein Target

The experiments were carried out to identify the gene product(s) of *A. fumigatus *targeted by the active molecule obtained from *E. coli. *The pathogenic *A. fumigatus *was cultured in the absence or presence of sublethal doses of the molecule. The proteins were isolated and separated on SDS gels for comparison of protein profile to identify the gene product affected by the molecule.

#### 2.7.1. Treatment of Pathogen with PPEBL21

The analysis of *A. fumigatus *proteins expressed differentially in untreated or that treated with antifungal protein PPEBL21 was carried out to identify gene products targeted by PPEBL21 as per the method described by Chhillar et al. [14]. The *A. fumigatus *was cultured in asparagine broth, a synthetic medium, which was prepared by dissolving asparagine (7.0 gm), ammonium chloride (7.0 gm), potassium dihydrogen phosphate (1.31 gm), ferric chloride (0.30 gm), dextrose (10.00 gm), sodium citrate (0.90 gm), and glycerol (25.0 ml) in 1000 ml of distilled water. The medium was dispensed into 250 ml flasks and sterilized at 10 psi for 15 minutes. The flasks were divided into two sets, the test and control. Sublethal doses of PPEBL21 were added to test set of the flasks. In the control set, only solvent was added. The flasks of both sets were inoculated with spores of *A. fumigatus *and incubated at 37°C in a BOD incubator for 36 hours.

#### 2.7.2. Protein Extraction

The cultures of treated and untreated flasks were harvested after 36 hours. The medium was removed and mycelial mat was inactivated by treating with 5% formaldehyde overnight. The fungal mat was dried at 37°C and lyophilized. It was crushed in mortar pestle to obtain fine powder. The fungal powder was defatted with diethylether with several changes until it became colorless. The suspension was then filtered through Whatman filter paper no. 1. It was then dried and stored in airtight bottles at −20°C for further use. The defatted fungal materials were extracted with 1 : 20 (w/v) 0.05 M ammonium bicarbonate buffer, pH 8.0 by continuous stirring for 24 hours [15]. The suspension was centrifuged at 16,500 g for 30 minutes at 4°C. The supernatant was dialyzed against water for 24 hours using membrane of 10 kDa cutoff. The extracts were again centrifuged and then passed through a Millipore filter membrane (0.45 *μ*m). The filtrates of control and treated pathogens were lyophilized and labeled properly before storage at −70°C. The proteins obtained from different cultures were estimated by BCA method and resolved by SDS-PAGE for comparison. The profile of protein bands of the mycelial proteins obtained from treated and untreated *A. fumigatus *were compared to identify the gene product(s) targeted by PPEBL21.

#### 2.7.3. Peptide Mass Fingerprinting by MALDI TOF MS

The protein band of *A. fumigatus *targeted by PPEBL21 was sliced out and subjected to in-gel digestion with trypsin. The proteolytic fragments separated on a microbore HPLC column were subsequently analyzed by LCMS as described by Gao et al. [16]. The Mascot algorithm was then employed to identify the peptide matches in candidate proteins available in MSDB database.

## 3. Results

The PPEBL21 having anti-*Aspergillus* and anti-Candidal property was subjected to the biological and biochemical characterization.

### 3.1. Elucidation of Isoelectric Point

The PPEBL21 was subjected to isoelectric focusing using IPG strips of 3–10 pH range to determine the isoelectric point. The results of two-dimensional electrophoresis demonstrated the pI of PPEBL21 to be 7.8.

### 3.2. Biochemical Properties 

#### 3.2.1. ADH Activity

**Figure 1:**
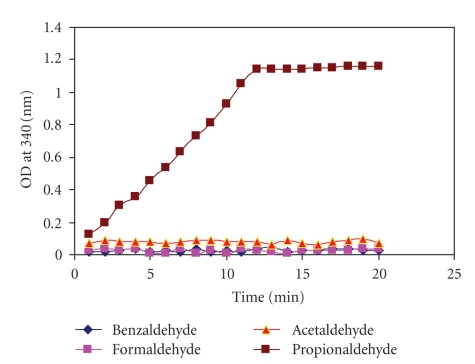
Enzyme activity of PPEBL21 using different aldehydes as substrate at 10 mM concentration. The ADH activity of PPEBL21 was propionaldehyde-specific.

PPEBL21 was examined for ADH activity using four different aldehydes as substrate. It was observed that PPEBL21 reduced propionaldehyde preferentially (Figure 1). The addition of propionaldehyde to the reaction mixture containing PPEBL21 resulted in formation of the yellowish product. The rate of product formation increased with increase in incubation period and reaction was completed within 13 minutes. The PPEBL21 did not show enzyme activity with other aldehydes as there was no product formation up to 20 minutes.

#### 3.2.2. In-Gel ADH Activity

**Figure 2:**
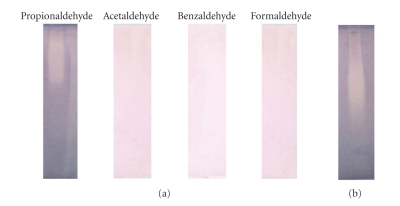
Propanol preferring ADH activity of PPEBL21 demonstrated by in-gel digestion assay. (a) PPEBL21 incubated with different aldehydes. (b) Standard ADH incubated with propionaldehyde.

PPEBL21 was further examined for in-gel ADH activity using four different aldehydes as substrate. Enzyme activity was detected by treating the gel with staining mixture containing 100 mM of aldehydes. A colourless smear was observed in-gels incubated with solution containing propionaldehyde as substrate (Figure 2). The gels which were treated with acetaldehyde, formaldehyde or benzaldehyde as substrate did not show any presence of ADH activity. The standard ADH (from *Saccharomyces cerevisiae*) procured from Sigma, USA, was used as positive control. The standard ADH also reduced the propionaldehyde and developed a colourless smear in the gel on incubation with staining mixture (Figure 2). Thus, the results reconfirmed that PPEBL21 had propionaldehyde-specific ADH activity.

#### 3.2.3. Enzyme Kinetics

**Figure 3:**
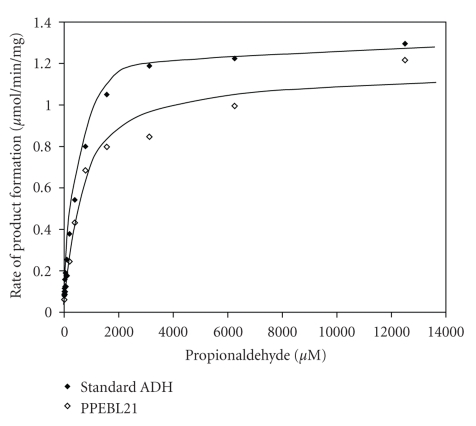
Estimation of Km and Vmax of PPEBL21 with Michaelis-Menten plot using propionaldehyde as substrate.

The reduction of propionaldehyde by PPEBL21 in the presence of NADH was investigated at different substrate concentrations. The kinetic properties of the purified PPEBL21 were determined using a software, namely, Sigma plot 8.0. Michaelis-Menten analysis indicated the dependence of product formation rate on substrate concentration. The Km and Vmax values of PPEBL21 were estimated to be 644.8 *μ*M and 1.2 U/mg, respectively, whereas standard ADH reduced propionaldehyde at faster rate and its Km was 481.9 *μ*M and Vmax was 1.3 U/mg (Figure 3).

### 3.3. Stability of PPEBL21

**Figure 4:**
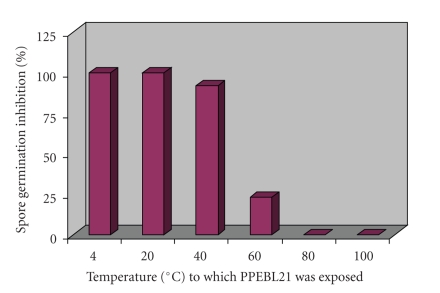
Percentage germination inhibition of *Aspergillus *spores by PPEBL21 exposed to various temperatures for 20 minutes.

The anti-*Aspergillus* activity of PPEBL21 after exposure to various temperatures and pH conditions was determined. The PPEBL21 was found to withstand the temperature of 40°C. The activity of PPEBL21 decreased with increasing temperature and protein was completely inactivated by heating at 80°C for 20 minutes in 10 mM Tris-HCl buffer at pH 7.5. Only 23% activity was demonstrated when PPEBL21 was heated up to 60°C (Figure 4). 

**Figure 5:**
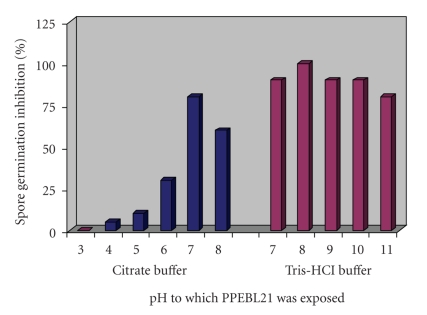
Percentage germination inhibition of *Aspergillus *spores by PPEBL21 exposed to various pHs for 20 minutes.

 PPEBL21 was found to be stable at pHs ranging from 7.0 to 10.5 and the maximal activity was observed at pH 7.0 to 8.0 in Tris-HCl buffer. In citrate phosphate buffer (pH 2.5 to 8.0) PPEBL21 was found to be less active and its maximal anti-*Aspergillus *activity (76.67%) was observed at pH 7.0 in citrate phosphate buffer (Figure 5).

### 3.4. Target Gene-Products

**Figure 6:**
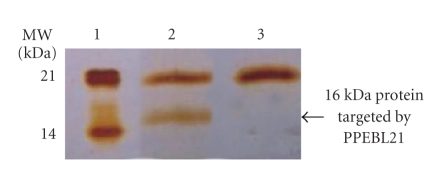
Protein profile of *A. fumigatus *treated with PPEBL21 for 36 hours. (Lane 1) Marker. (Lane 2) Control. (Lane 3) Treated with PPEBL21.

The mycelial proteins of untreated and treated cultures of *A. fumigatus *after 36 hours were analyzed to determine the effect of the molecule on gene products of the pathogen. The protein concentration in mycelial extract was determined and equal amounts of both proteins (untreated and treated) were run on SDS-PAGE. Figure 6 shows the effect of PPEBL21 on protein profile of *A. fumigatus. *It was observed that the expression of one protein in the molecular weight range of 16.0 kDa was completely inhibited by the molecule. These results indicated that the gene of this protein was the potential target for PPEBL21.

 The protein which was targeted by PPEBL21 was sliced out from the gel and subjected for analysis by LCMS. As a result the sequence of the protein was found to be IENINGEFVFH. This sequence of the protein was subjected to MASCOT search for sequence homology. The best match of the sequence obtained in the current study showed a score of 64 with a hypothetical protein of *A. fumigatus. *It is, therefore, presumed that the gene responsible for expression of 16.0 kDa hypothetical protein in *A. fumigatus *may be one of the important targets for PPEBL21.

## 4. Discussion

Since MALDI and N-Terminal amino acid sequence results suggested that PPEBL21 might be an alcohol dehydrogenase from medium chain dehydrogenase family [10], we investigated the molecule for its ADH activity. Initial enzyme activity experiments showed that PPEBL21 was able to catalyze the conversion of specific aldehyde substrate into colored product. We observed that at pH 7.5, PPEBL21 in the presence of NADH, reduced propionaldehyde only but it was not able to reduce other aldehydes. The optimum activity of ADH using propionaldehyde as a substrate was observed by MacGibbon et al. [17] also at pH 7.6. Brisdelli et al. [18] investigated ADH purified from *K. lactis* for its activity using various aldehydes such as acetaldehyde, propionaldehyde, butyraldehyde, formaldehyde, glutaraldehyde, salicylaldehyde, cinnamaldehyde, cyclohexanecarbaldehyde, and benzaldehyde and found that their preparation was able to reduce propionaldehyde and butyraldehyde only. Schenkels and Duine [19] purified an ADH from *Rhodococcus erythropolis* DSM 1069 which could reduce formaldehyde only but not other tested aldehydes. Bryant et al. [20] reported ADH from *Thermoanaerobacter ethanolicus *which was able to reduce propionaldehyde. These observations as well as the results obtained in the present study suggested that the ADH-like molecules obtained from different sources may differ in their specificity for the substrates. 

 The substrate specificity of an enzyme is generally determined by the size, shape and location of substrate cleft. The substrate binding site of ADH has been found to be a 20 Å deep cleft [21]. The inner part of the substrate cleft is formed by the catalytic zinc at the bottom, its ligand and the nicotinamide ring of the coenzyme. The main part of the substrate cleft has been very hydrophobic because of the amino acid side chains forming the lining which determines the substrate specificity [22]. Horse liver ADH was reported to oxidize and reduce wide range of alcohols and aldehydes. It oxidized some secondary alcohols also probably due to the large size of substrate cleft and the presence of smaller residues in the walls of the pocket compared with other ADHs [20]. The reduced size of amino acids may allow the enhanced reduction of aldehydes larger than acetaldehyde. It has been shown that ADHs containing Ser rather than Thr at position 48 were able to reduce larger aldehydes rather than smaller [23]. It might be possible that PPEBL21 contains compatible size of active cleft having small size amino acids like Ser at appropriate position in the reactive pocket which enabled it to reduce propionaldehyde most optimally.

 In-gel digestion assay also proved that PPEBL21 was an alcohol dehydrogenase. After native PAGE and subsequent staining with propionaldehyde, PPEBL21 activity was found as smear but this activity was not detected when other aldehydes were used as substrate. When standard ADH (purchased from Sigma) was incubated with propionaldehyde, it showed ADH activity by reducing the propionaldehyde in the presence of NADH. Vaglenova et al. [24] also observed smear when purified ADH was incubated with staining mixture alongwith substrate, 1-butanol. Similarly, Hou et al. [25] reported ADH activity by incubating the ADH containing native gel in primary and secondary butanol as substrate. The kinetic data of PPEBL21 plotted according to Michaelis-Menten, resulted in typical curves indicating that the enzyme followed a sequentially ordered mechanism of rate of reaction [26] which has been described for an ADH [27]. 

 Iso-electric point of PPEBL21 was found to be 7.8, but pI of propanol preferring ADH of *E. coli* has been reported to be 5.1 [28]. It is thus hypothesized that PPEBL21 isolated from *E. coli *BL21 might contain some specific sites in the molecule for ADH activity but it could be a different molecule than ADH. The difference, although very small, in molecular weight of PPEBL21 from that of ADH of *E. coli *reported earlier [27], was also evident. Since the PPEBL21 demonstrated sequence similarity with ADH and also the ADH activity with specific substrates, we had reason to believe that PPEBL21 was a molecule of ADH family. So, an immediate question was that if PPEBL21 was an ADH, then commercially available ADH may contain antifungal activity. But surprisingly we did not find antifungal activity in commercially available ADH (of *Saccharomyces cerevisiae*) that we purchased from Sigma Chemical Co. USA. It thus, appears that PPEBL21 is a unique molecule which has potential against fungi pathogenic to humans and also has the active sites responsible for ADH activity. 

 In the present study, we found that antifungal activity of PPEBL21 remained intact up to 40°C and there was complete loss of inhibitory activity at 80°C. Kim and Chung [28] reported that antifungal activity of protein purified from *B. amyloliquifaciens *MET0908 was stable up to 70°C without significant loss of activity but showed sensitivity to increased temperatures. It demonstrated 70% activity after exposure to a temperature of 80°C and only 50% activity was observed after exposure of their protein to 100°C. The antifungal activity of SAP purified from *Streptomyces* was reported to be decreased with increasing temperature and lost its activity completely at 90°C [29]. Magnusson and Schnurer [30] recovered comparatively less stable antifungal products because they observed that the antifungal activity of freeze dried culture was lost during prolonged storage. Woo et al. [29] demonstrated that SAP was active over a wide pH range starting from pH 6.0 to pH 11.0 and the maximal activity was observed between pH 7.0 to 8.0. We also observed the maximal activity of PPEBL21 at pH 7.0 to 8.0. 

 The molecule PPEBL21 was also studied to find out its possible targets in the pathogens. The analysis of protein profile from treated and untreated *A. fumigatus *showed that the molecule completely inhibited the expression of few genes at the time of early development. One of the major proteins of *A. fumigatus *which was found to be completely inhibited by the molecule was 16 kDa. The analysis of peptide mass fingerprinting and homology search indicated that the protein targeted by PPEBL21 showed a significant score of 64 with hypothetical protein of *A. fumigatus*. More work is required to establish if the gene product targeted by PPEBL21 was hypothetical protein of *A. fumigatus*. However, the information obtained from the results suggested that the inhibition of the hypothetical protein may be useful to develop high throughput screening system for screening of new molecules.
